# Supplementary material for: Exploring structure-function relationships in engineered receptor performance using computational structure prediction
Source: bioRxiv. 2024 Nov 7:2024.11.07.622438. Preprint. [Version 1] doi: 10.1101/2024.11.07.622438 (PMC11581020; doi:10.1101/2024.11.07.622438)
Supplement: Supplement 1 — Supplementary material online includes Supplementary Information (Supplementary Figures 1-9, Supplementary Tables 1, 2, 3), Supplementary Data 1 (archive containing ColabFold-generated structural models used in this study) and Supplementary Data 2 (archive containing PREDDIMER-generated TMD dimers used in this study)49, and a Source Data file containing values used to generate all plots reported. [file media-1.pdf]

Supplementary Information for

Exploring structure-function relationships in engineered receptor performance using computational structure prediction

William K. Corcoran[1,2,3], Amparo Cosio[1,3], Hailey I. Edelstein[1,3], Joshua N. Leonard[1,2,3,4,5,*]

[1]Department of Chemical and Biological Engineering, Northwestern University, Evanston, Illinois 60208, United States

[2]Interdisciplinary Biological Sciences Program, Northwestern University, Evanston, Illinois 60208, United States

[3]Center for Synthetic Biology, Northwestern University, Evanston, Illinois 60208, United States

[4]Chemistry of Life Processes Institute, Northwestern University, Evanston, Illinois 60208, United States

[5]Member, Robert H. Lurie Comprehensive Cancer Center, Northwestern University, Evanston, Illinois 60208, United States

Contact Information:
*Corresponding author: j-leonard@northwestern.edu

Contents:

Supplementary Figures 1-9
Supplementary Tables 1-3

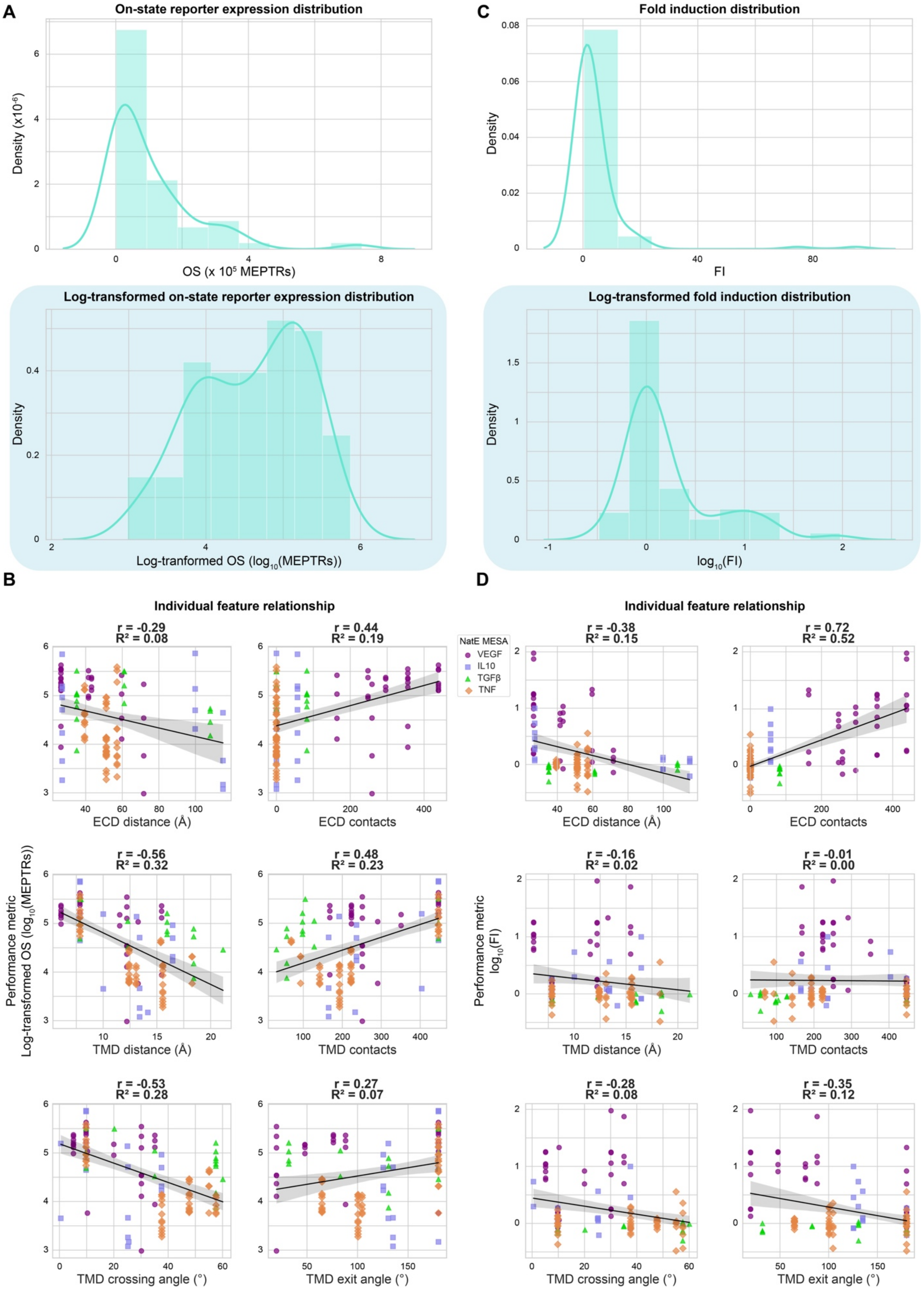
A
On-state reporter expression distribution
Density (x10-6)
OS (x 105 MEPTRs)
Log-transformed on-state reporter expression distribution
Density
Log-tranformed OS (log10(MEPTRs))
C
Fold induction distribution
Density
FI
Log-transformed fold induction distribution
Density
log10(FI)
B
Individual feature relationship
r = -0.29
R² = 0.08
ECD distance (Å)
r = 0.44
R² = 0.19
ECD contacts
NatE MESA
VEGF
IL10
TGFβ
TNF
Performance metric
Log-transformed OS (log10(MEPTRs))
r = -0.56
R² = 0.32
TMD distance (Å)
r = 0.48
R² = 0.23
TMD contacts
r = -0.53
R² = 0.28
TMD crossing angle (°)
r = 0.27
R² = 0.07
TMD exit angle (°)
D
Individual feature relationship
r = -0.38
R² = 0.15
ECD distance (Å)
r = 0.72
R² = 0.52
ECD contacts
Performance metric
log10(FI)
r = -0.16
R² = 0.02
TMD distance (Å)
r = -0.01
R² = 0.00
TMD contacts
r = -0.28
R² = 0.08
TMD crossing angle (°)
r = -0.35
R² = 0.12
TMD exit angle (°)

Supplementary Figure 1: Identifying structural features that can explain global trends in MESA performance

(**A**) All four datasets (VEGF MESA, TNF MESA, IL-10 MESA, TGFβ MESA) were aggregated into a single "all receptor" dataset, with the OS distribution (top) and log-transformed OS (bottom) with fitted kernel density estimate (KDE). Log-transformed OS was selected as the performance metric for further analysis as the OS distribution is right-skewed. (**B**) Evaluation of the linear relationship between each individual structural feature and log-transformed OS. (**C**) The "all receptor" FI distribution (top) and log-transformed FI distribution (bottom) with fitted KDEs. Log-transformed FI was selected as the performance metric for further analysis as the OS distribution has a greater right skew. (**D**) Evaluation of the linear relationship between each individual structural feature and log-transformed FI. Abbreviations: on-state reporter expression (OS), fold induction (FI), kernel density estimate (KDE), molecules of equivalent PE-TexasRed (MEPTRs), adjusted R^2 (Adj. R^2), Pearson's correlation coefficient (r), ectodomain (ECD), transmembrane domain (TMD), natural ectodomain modular extracellular sensor architecture (NatE MESA), vascular endothelial growth factor (VEGF), interleukin 10 (IL-10), transforming growth factor β (TGFβ), tumor necrosis factor (TNF).

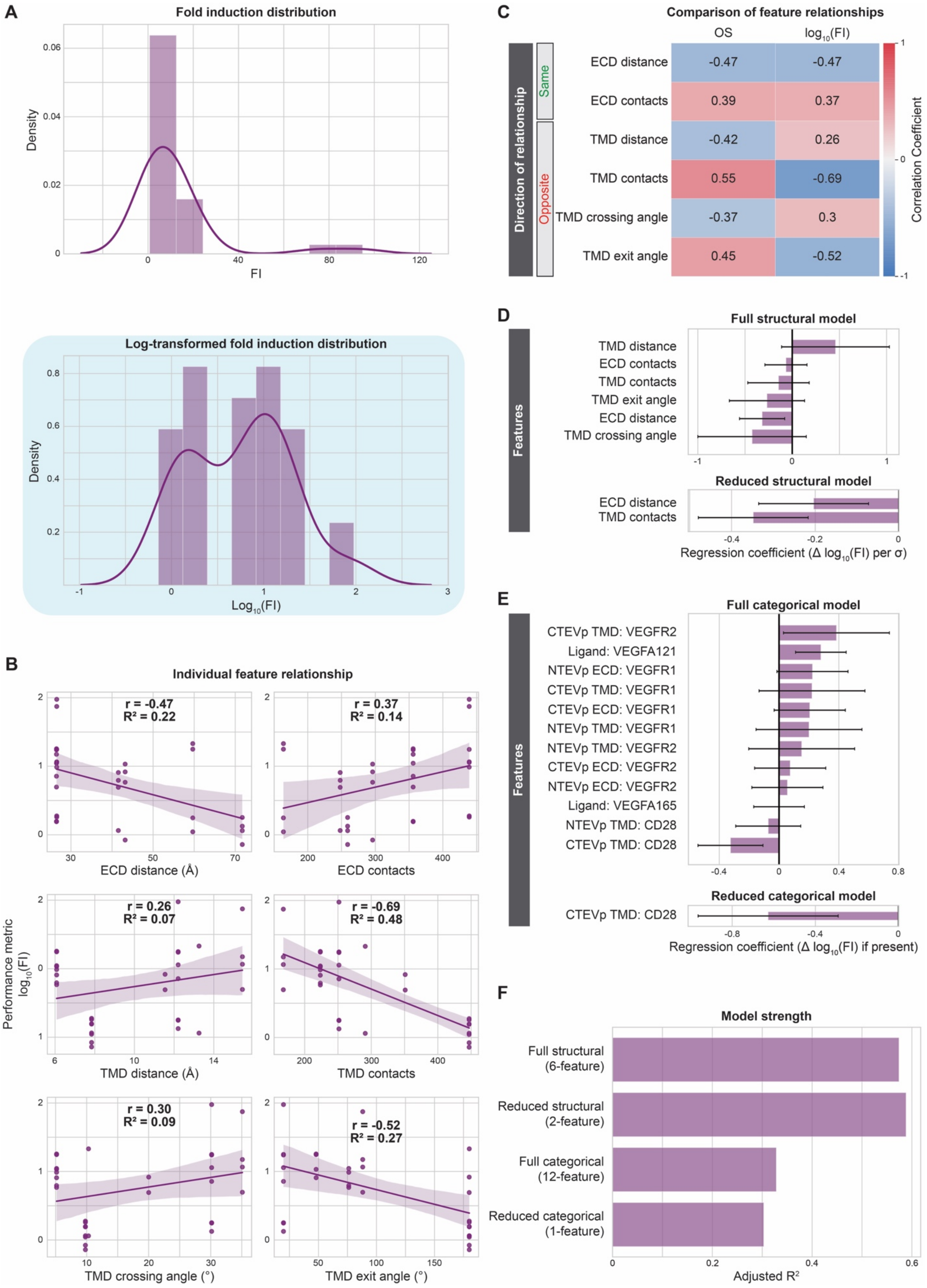
A
Fold induction distribution
Density
FI
Log-transformed fold induction distribution
Density
Log10(FI)
B
Individual feature relationship
Performance metric log10(FI)
r = -0.47
R² = 0.22
ECD distance (Å)
r = 0.37
R² = 0.14
ECD contacts
r = 0.26
R² = 0.07
TMD distance (Å)
r = -0.69
R² = 0.48
TMD contacts
r = 0.30
R² = 0.09
TMD crossing angle (°)
r = -0.52
R² = 0.27
TMD exit angle (°)
C
Comparison of feature relationships
OS
log10(FI)
Direction of relationship
Same
Opposite
ECD distance -0.47 -0.47
ECD contacts 0.39 0.37
TMD distance -0.42 0.26
TMD contacts 0.55 -0.69
TMD crossing angle -0.37 0.3
TMD exit angle 0.45 -0.52
Correlation Coefficient
D
Full structural model
Features
TMD distance
ECD contacts
TMD contacts
TMD exit angle
ECD distance
TMD crossing angle
Reduced structural model
ECD distance
TMD contacts
Regression coefficient (Δ log10(FI) per σ)
E
Full categorical model
Features
CTEVp TMD: VEGFR2
Ligand: VEGFA121
NTEVp ECD: VEGFR1
CTEVp TMD: VEGFR1
CTEVp ECD: VEGFR1
NTEVp TMD: VEGFR1
NTEVp TMD: VEGFR2
CTEVp ECD: VEGFR2
NTEVp ECD: VEGFR2
Ligand: VEGFA165
NTEVp TMD: CD28
CTEVp TMD: CD28
Reduced categorical model
CTEVp TMD: CD28
Regression coefficient (Δ log10(FI) if present)
F
Model strength
Full structural (6-feature)
Reduced structural (2-feature)
Full categorical (12-feature)
Reduced categorical (1-feature)
Adjusted R²

Supplementary Figure 2: Structural features account for considerable variation in VEGF MESA fold induction

(**A**) The distribution of VEGF MESA FIs (top) and log-transformed FIs (bottom) with a fitted kernel density estimate (KDE). Given the right skew of the FI distribution, we selected log-transformed FI as the performance metric for further analysis. (**B**) Evaluation of the linear relationship between each individual structural feature and log-transformed FI. (**C**) Comparison between the sign of the relationship between each structural feature with OS and $\log_{10}$(FI) as the performance metric. Both ECD metrics correlate in the same direction for either performance metric, while all four TMD features correlate in opposite directions with the two performance metrics. (**D**) Magnitude of the coefficients for the full (six-feature) and reduced (two-feature) structural models. Error bars represent +/- 95% confidence intervals calculated using Student's t-distribution. (**E**) Magnitude of the coefficients for the full (twelve-feature) and reduced (two-feature) categorical models. Error bars represent +/- 95% confidence intervals calculated using Student's t-distribution. (**F**) Comparison of the four models' strengths in explaining variation in OS, as determined by adjusted R^2. Abbreviations: vascular endothelial growth factor receptor (VEGFR), vascular endothelial growth factor receptor 1 (VEGFR1), vascular endothelial growth factor receptor 2 (VEGFR2), vascular endothelial growth factor (VEGF), adjusted R^2 (Adj. R^2), Pearson's correlation coefficient (r), coefficient of determination (R^2), on-state reporter expression (OS), fold induction (FI), ectodomain (ECD), transmembrane domain (TMD), cluster of differentiation 28 (CD28), N-terminal half of Tobacco Etch Virus Protease (NTEVp), C-terminal half of Tobacco Etch Virus Protease (CTEVp), change in (Δ), standard deviation (σ).

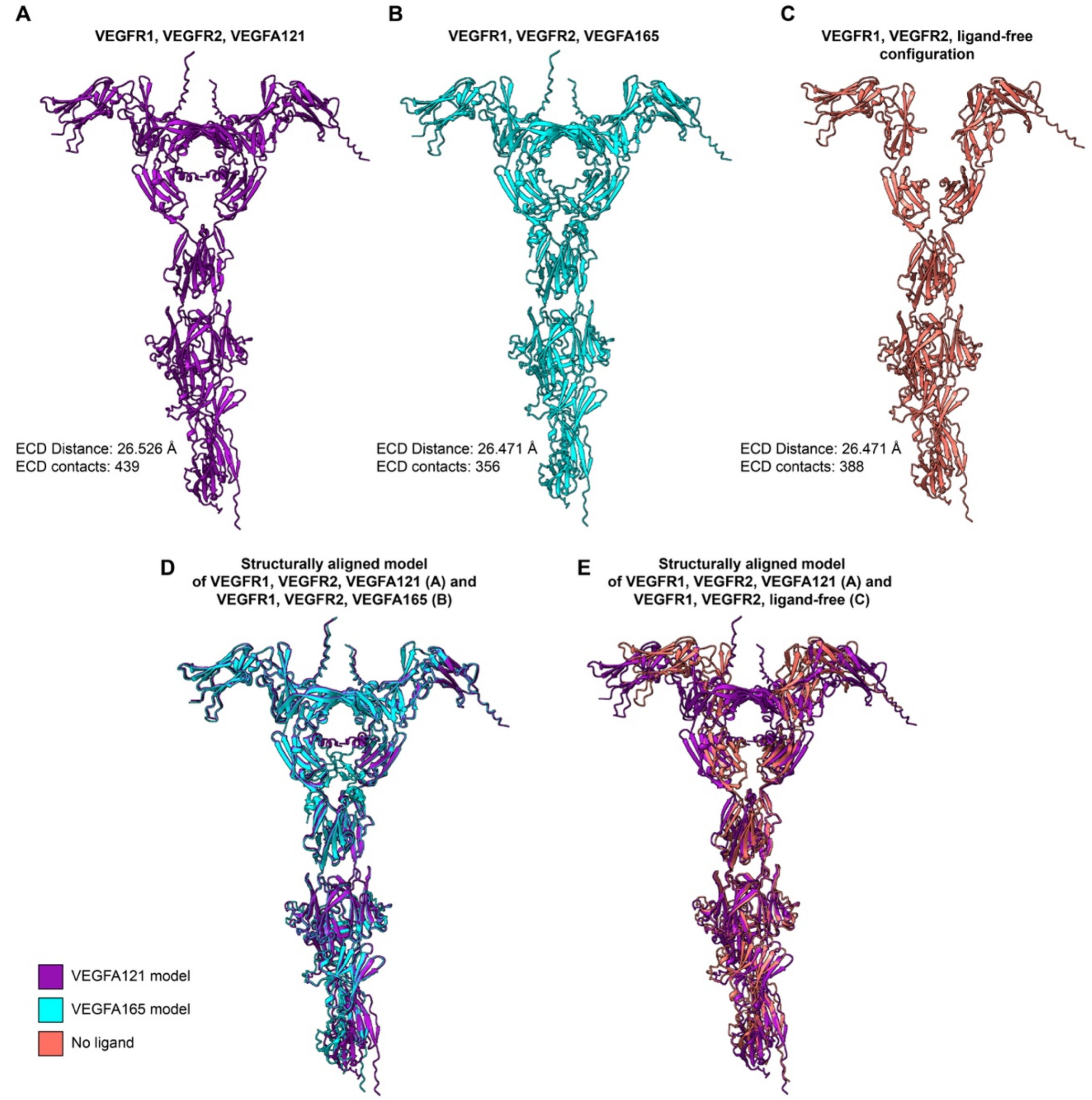

Supplementary Figure 3: Comparison of ColabFold-generated VEGFR ECD models
(**A**) Structural model of VEGFR1 and VEGFR2 in complex with a VEGFA121 dimer (**Supplementary Data 1, Structure 6**). (**B**) Structural model of VEGFR1 and VEGFR2 in complex with a VEGFA165 dimer (**Supplementary Data 1, Structure 7**). (**C**) Structural model of VEGFR1 and VEGFR2 dimerized in the absence of ligand (apo configuration) (**Supplementary Data 1, Structure 19**). (**D**) Superimposition of the structural model in **A** and **B**, wherein the difference in the structures is the ligand: VEGFA121 or VEGFA165. The structures have a high degree of similarity, resulting in similar measurements for their ECD features. (**E**) Superimposition of the structural model in **A** and **C**, wherein the difference in the structures is the ligand: VEGFA121 or apo (no ligand). The structures have a high degree of similarity, resulting in highly similar measurements for their ECD features. Abbreviations: vascular endothelial growth factor receptor 1 (VEGFR1), vascular endothelial growth factor receptor 2 (VEGFR2), vascular endothelial growth factor A, 121 amino acid isoform (VEGFA121), vascular endothelial growth factor A, 165 amino acid isoform (VEGFA165).

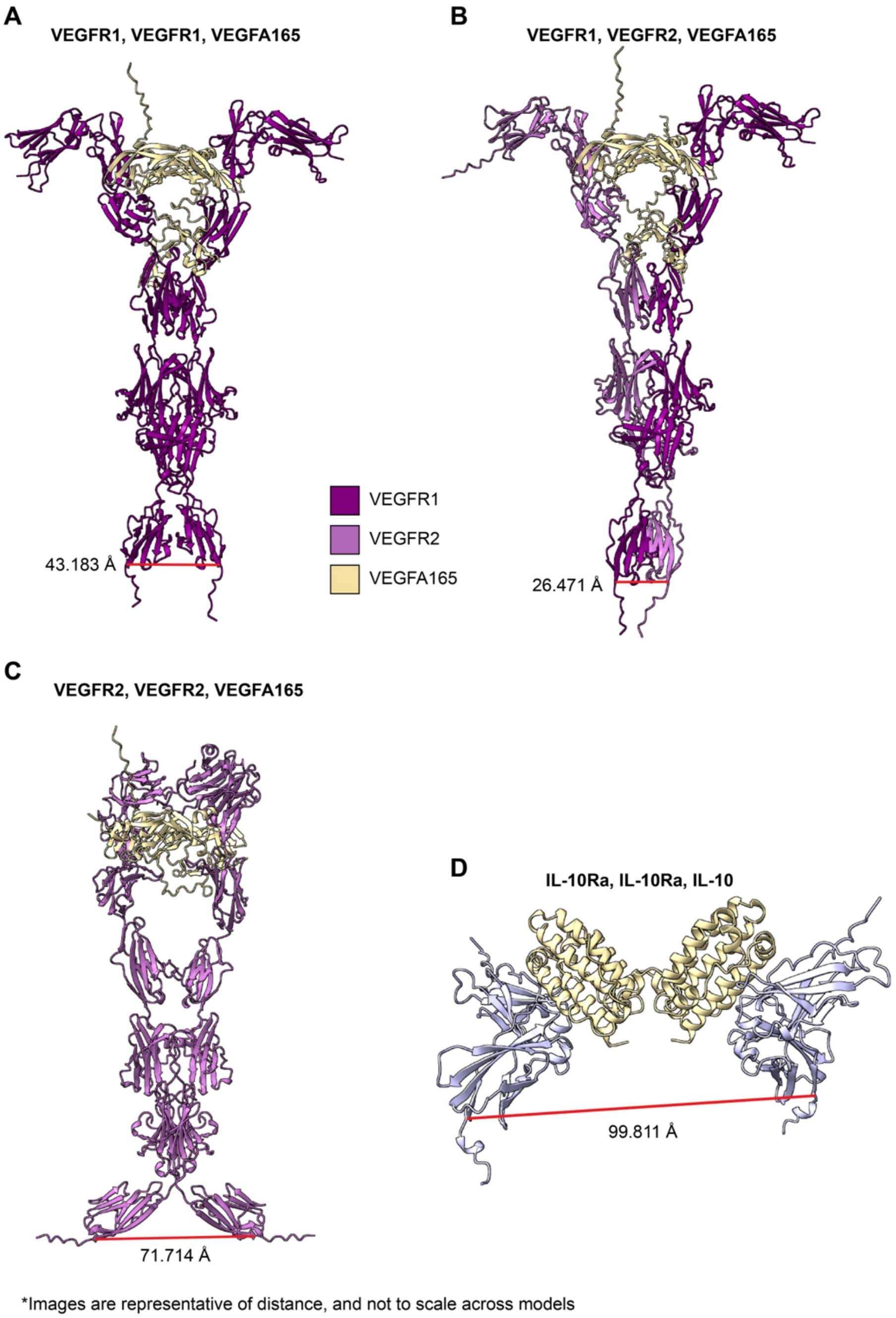

Supplementary Figure 4: ECD distance varies considerably between ColabFold-generated models
(**A**) ECD distance measurement of the structural model for VEGFR1 and VEGFR1 in complex with a VEGFA165 dimer (**Supplementary Data 1, Structure 5**). (**B**) ECD distance measurement of the structural model for VEGFR1 and VEGFR2 in complex with a VEGFA165 dimer (**Supplementary Data 1, Structure 7**). (**C**) ECD distance measurement of the structural model for VEGFR2 and VEGFR2 in complex with a VEGFA165 dimer (**Supplementary Data 1, Structure 9**). (**D**) ECD distance measurement of the structural model for IL-10Ra and IL-10Ra in complex with an IL-10 dimer (**Supplementary Data 1, Structure 13**). Abbreviations: ectodomain (ECD), vascular endothelial growth factor receptor 1 (VEGFR1), vascular endothelial growth factor receptor 2 (VEGFR2), vascular endothelial growth factor A, 121 amino acid variant (VEGFA121), vascular endothelial growth factor A, 165 amino acid variant (VEGFA165), interleukin 10 receptor a (IL-10Ra), interleukin 10 (IL-10).

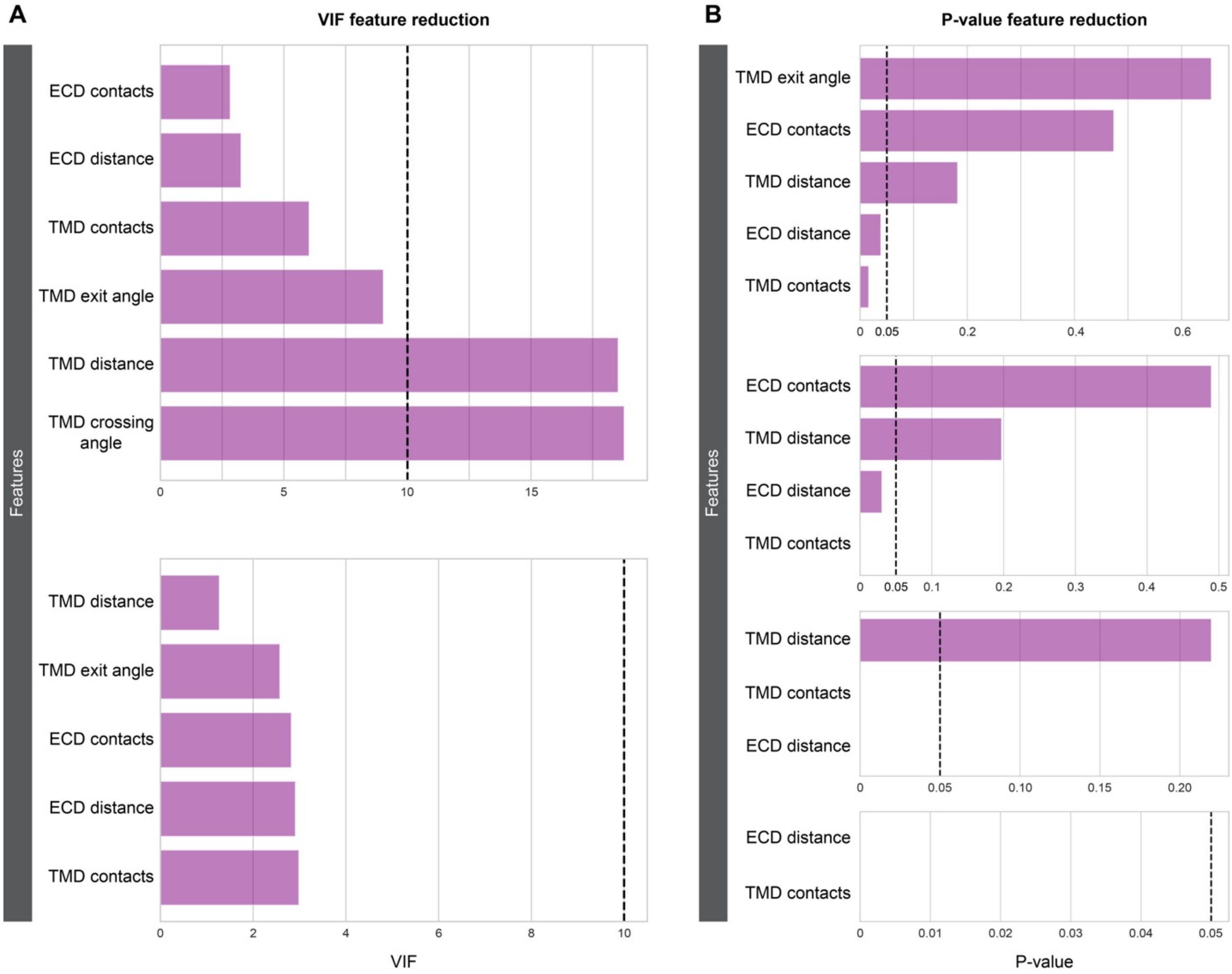

Supplementary Figure 5: **VEGFR structural model reduction methodology**
(**A**) Variance inflation factor (VIF) calculation for all six structural features (top). Removing TMD crossing angle resulted in lowering the VIF for all features below the threshold of 10. (**B**) Backwards elimination for the remaining five structural features (top), removing one feature each round based upon the coefficient p-value ($\alpha = 0.05$), until only statistically significant features remain (bottom). Abbreviations: variance inflation factor (VIF), ectodomain (ECD), transmembrane domain (TMD).

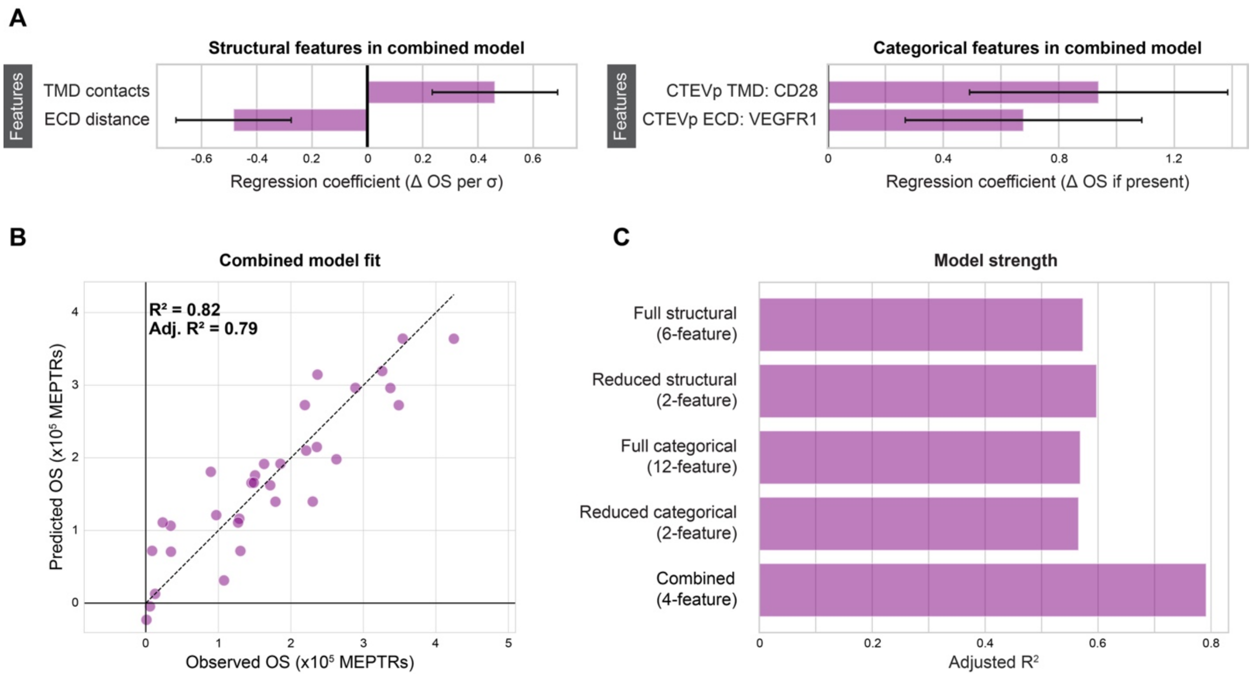

Supplementary Figure 6: VEGF MESA combined model improves explanatory strength

(**A**) Magnitude of the structural coefficients (left) and the categorical features (right) in the combined (four-feature) model. Error bars represent +/- 95% confidence intervals calculated using Student's t-distribution. (**B**) Combined (four-feature) model fit to explain variation in VEGF MESA OS. (**C**) Comparison of the four models' strengths in explaining variation in OS, as determined by adjusted R^2. Combining the features from the reduced structural model and reduced categorical model yielded a combined model with greater explanatory strength, indicating that the structural features and categorical features do not directly encode the same information to explain variation in OS. Abbreviations: transmembrane domain (TMD), ectodomain (ECD), C-terminal half of Tobacco Etch Virus Protease (CTEVp), cluster of differentiation 28 (CD28), adjusted R^2 (Adj. R^2), Pearson's correlation coefficient (r), coefficient of determination (R^2), on-state reporter expression (OS), molecules of equivalent PE-TexasRed (MEPTRs), change in (Δ), standard deviation (σ).

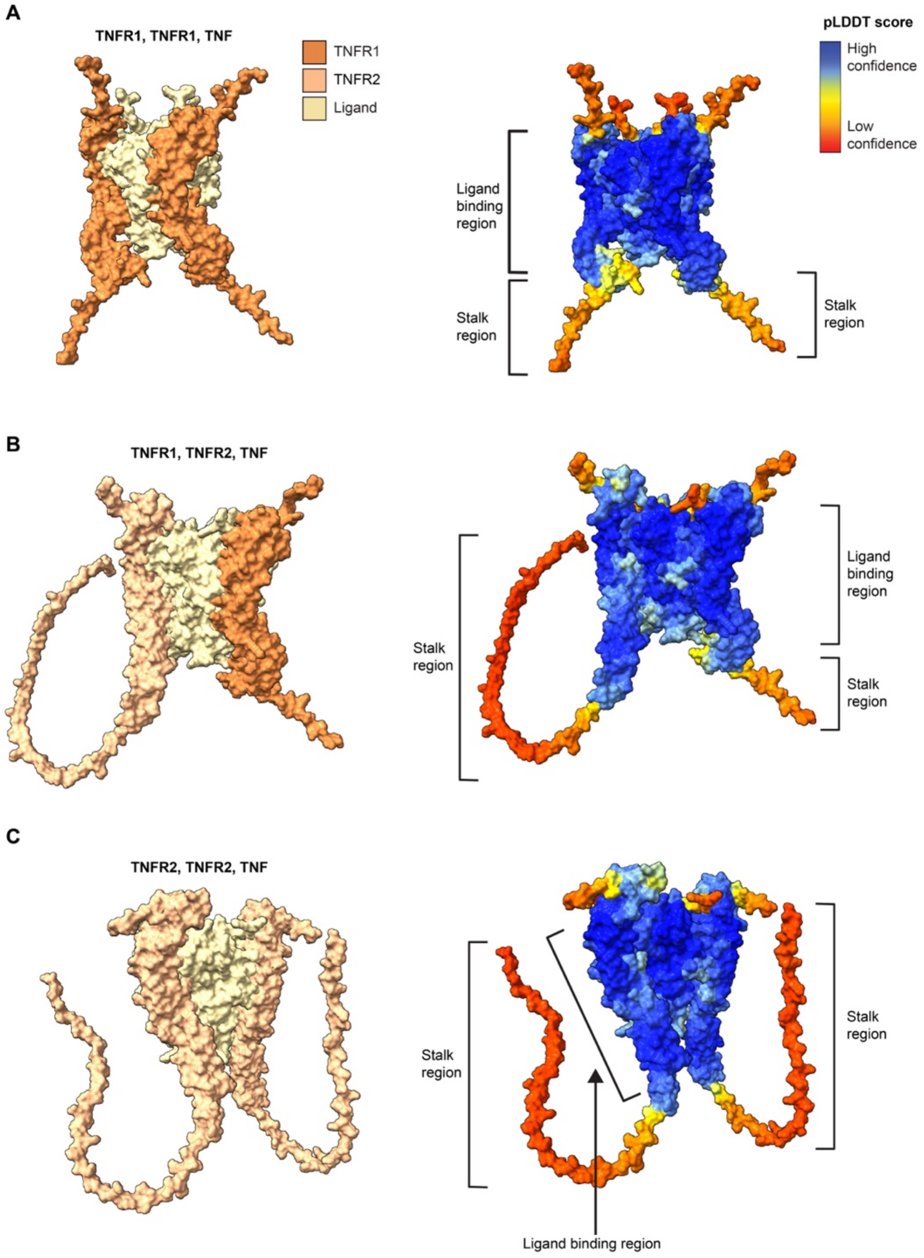

Supplementary Figure 7: TNFR stalk regions are unstructured and have low confidence scores
(**A**) Structural model of a TNFR1 and TNFR1 in complex with TNF colored by protein (left) and confidence score (right) (**Supplementary Data 1, Structure 10**). The stalk regions have low confidence scores. (**B**) Structural model of a TNFR1 and TNFR2 in complex with TNF colored by protein (left) and confidence score (right) (**Supplementary Data 1, Structure 11**). The stalk regions have low confidence scores and the TNFR2 stalk region folds upward, toward the ligand-binding region. (**C**) Structural model of a TNFR2 and TNFR2 in complex with TNF colored by protein (left) and confidence score (right) (**Supplementary Data 1, Structure 12**). The stalk regions have low confidence scores and both TNFR2 stalk region folds upward, toward the ligand-binding region. Abbreviations: predicted local distance difference test (pLDDT), tumor necrosis factor receptor (TNFR), tumor necrosis factor receptor 1 (TNFR1), tumor necrosis factor receptor 2 (TNFR2), tumor necrosis factor (TNF).

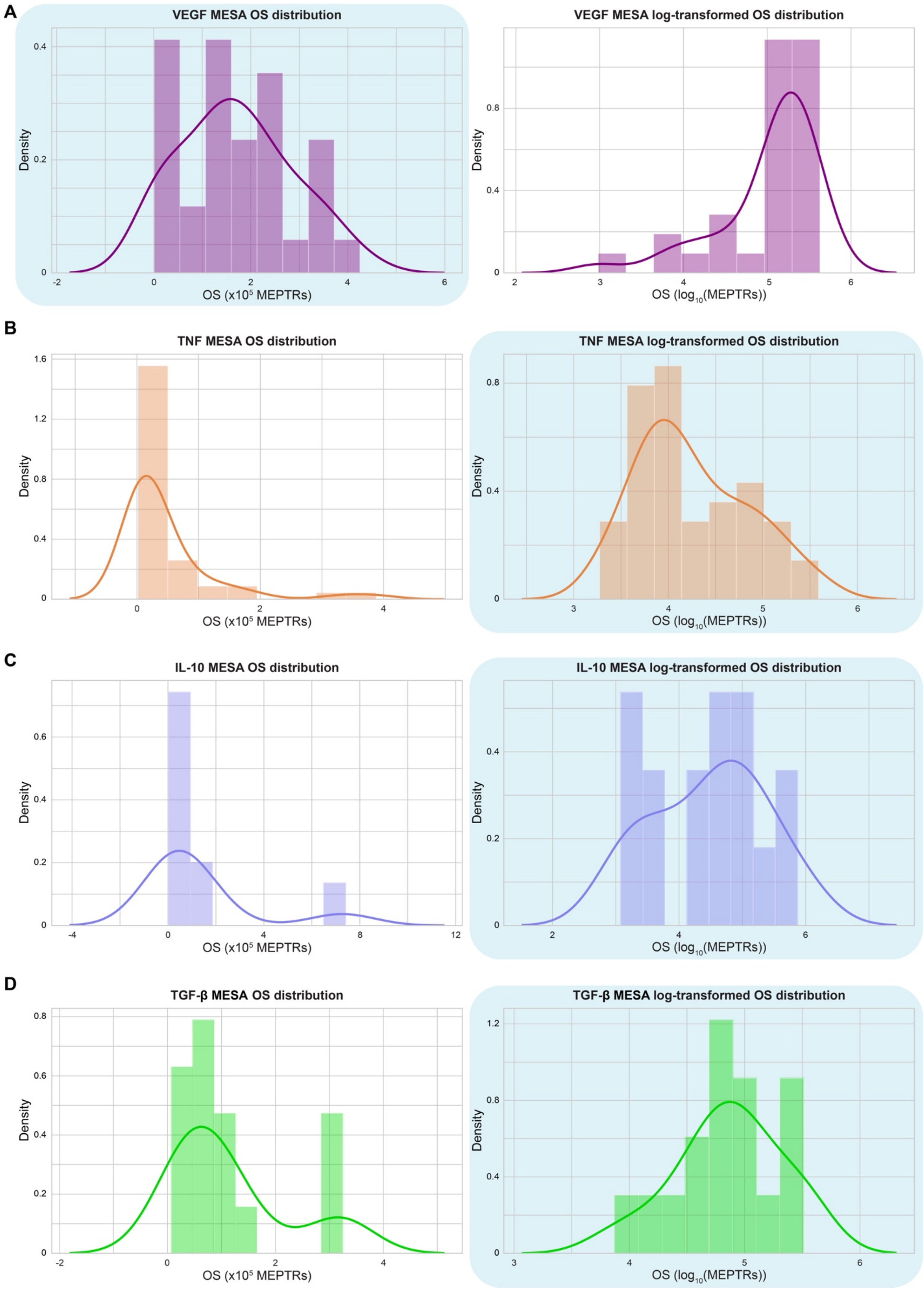
A
VEGF MESA OS distribution
Density
OS (x10^5 MEPTRs)
VEGF MESA log-transformed OS distribution
OS (log10(MEPTRs))
B
TNF MESA OS distribution
TNF MESA log-transformed OS distribution
C
IL-10 MESA OS distribution
IL-10 MESA log-transformed OS distribution
D
TGF-β MESA OS distribution
TGF-β MESA log-transformed OS distribution

Supplementary Figure 8: **Selection of OS distributions**

(**A**) Distribution of VEGF MESA OS (left) and log-transformed OS (right) with fitted kernel density estimate (KDE). OS was selected as the performance metric for further analysis as log-transformed OS is left-skewed. (**B**) Distribution of TNF MESA OS (left) and log-transformed OS (right) with fitted KDEs. Log-transformed OS was selected as the performance metric for further analysis as OS is right-skewed. (**C**) Distribution of IL-10 MESA OS (left) and log-transformed OS (right) with fitted KDEs. Log-transformed OS was selected as the performance metric for further analysis as OS is right-skewed. (**D**) Distribution of TGFβ MESA OS (left) and log-transformed OS (right) with fitted KDEs. Log-transformed OS was selected as the performance metric for further analysis as OS is right-skewed. Abbreviations: on-state reporter expression (OS), vascular endothelial growth factor (VEGF), tumor necrosis factor (TNF), interleukin 10 (IL-10), transforming growth factor β (TGFβ), modular extracellular sensor architecture (MESA), ectodomain (ECD), transmembrane domain (TMD), molecules of equivalent PE-TexasRed (MEPTRs).

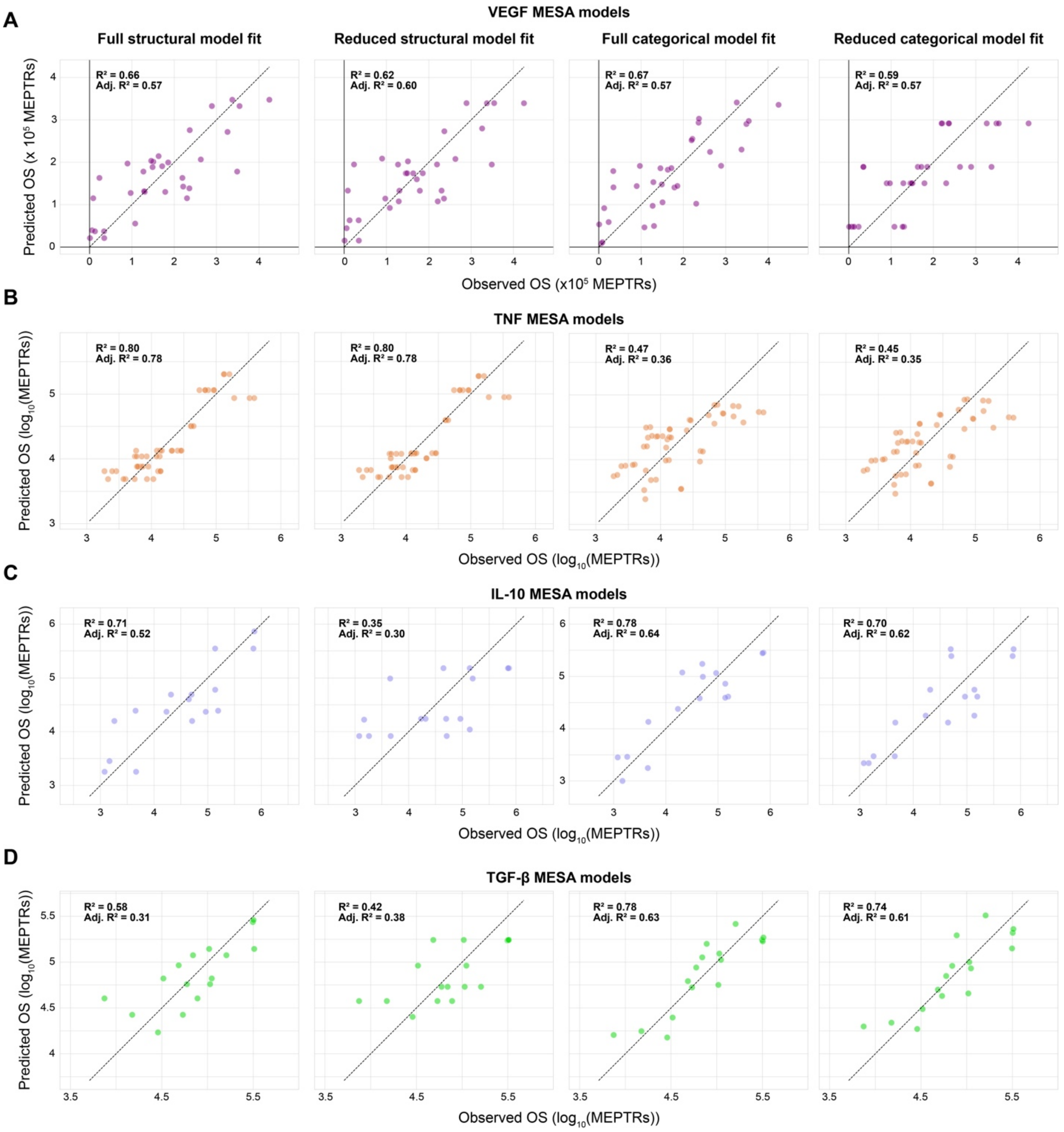

Supplementary Figure 9: Comparison between structural and categorical model fit

(**A**) Comparison between the VEGF MESA full structural model (six-feature) (left), reduced structural model (two-feature) (center left), full categorical model (twelve-feature) (center right), and the reduced categorical model (two-feature) (right) fit to explain variation in OS. (**B**) Comparison between the TNF MESA full structural model (six-feature) (left), reduced structural model (three-feature) (center left), full categorical model (thirteen-feature) (center right), and the reduced categorical model (eleven-feature) (right) fit to explain variation in log-transformed OS. (**C**) Comparison between the IL-10 MESA full structural model (six-feature) (left), reduced structural model (one-feature) (center left), full categorical model (ten-feature) (center right), and the reduced categorical model (five-feature) (right) fit to explain variation in log-

transformed OS. (**D**) Comparison between the TGFβ MESA full structural model (six-feature) (left), reduced structural model (one-feature) (center left), full categorical model (ten-feature) (center right), and the reduced categorical model (eight-feature) (right) fit to explain variation in log-transformed OS. Abbreviations: on-state reporter expression (OS), vascular endothelial growth factor (VEGF), tumor necrosis factor (TNF), interleukin 10 (IL-10), transforming growth factor β (TGFβ), modular extracellular sensor architecture (MESA), ectodomain (ECD), transmembrane domain (TMD), Pearson's correlation coefficient (r), adjusted R^2 (Adj. R^2), coefficient of determination (R^2), molecules of equivalent PE-TexasRed (MEPTRs).

Supplementary Table 1: ColabFold-generated structural models and confidence scores used in this study[1].

Model ID[2]	Receptor chains	Ligand	pLDDT score	pTM score	ipTM score
Structure 1	VEGFR2 NTEVp, VEGFR1 CTEVp	VEGFA165 (dimer)	72.2	0.601	0.522
Structure 2	IL-10Rb NTEVp, IL-10Ra CTEVp	IL-10 (dimer)	85.8	0.702	0.688
Structure 3	IL-10Rb NTEVp (CD28 TMD), IL-10Ra CTEVp (CD28TMD)	IL-10 (dimer)	80.5	0.656	0.652
Structure 4	VEGFR1, VEGFR1	VEGFA121 (dimer)	85.8	0.703	0.689
Structure 5	VEGFR1, VEGFR1	VEGFA165 (dimer)	85.2	0.682	0.678
Structure 6	VEGFR1, VEGFR2	VEGFA121 (dimer)	84.2	0.678	0.663
Structure 7	VEGFR1, VEGFR2	VEGFA165 (dimer)	82.2	0.665	0.658
Structure 8	VEGFR2, VEGFR2	VEGFA121 (dimer)	81.8	0.638	0.62
Structure 9	VEGFR2, VEGFR2	VEGFA165 (dimer)	80.1	0.636	0.628
Structure 10	TNFR1, TNFR1	TNF (trimer)	89.8	0.899	0.885
Structure 11	TNFR1, TNFR2	TNF (trimer)	87	0.87	0.851
Structure 12	TNFR2, TNFR2	TNF (trimer)	85.1	0.85	0.827
Structure 13	IL-10Ra, IL-10Ra	IL-10 (dimer)	91.4	0.869	0.865
Structure 14	IL-10Ra, IL-10Ra, IL-10Rb, IL-10Rb	IL-10 (dimer)	89.1	0.853	0.855
Structure 15	IL-10Rb, IL-10Rb	IL-10 (dimer)	91.5	0.78	0.74
Structure 16	TGFβR1, TGFβR1	TGF-β (dimer)	80.9	0.737	0.676
Structure 17	TGFβR1, TGFβR1, TGFβR2, TGFβR2	TGF-β (dimer)	84.1	0.787	0.759
Structure 18	TGFβR2, TGFβR2	TGF-β (dimer)	81.4	0.633	0.571
Structure 19	VEGFR1, VEGFR2	apo (no ligand)	82.2	0.599	0.608

1. Abbreviations: Model identification (Model ID), ectodomains (ECDs), predicted local distance difference test (pLDDT), predicted template modeling (pTM), interfacial predicted template modeling (ipTM)
2. Individual model files are included in supporting materials online as **Supplementary Data 1**.

Supplementary Table 2: Peptide sequences used in this study.

Sequence ID	Description: ECD, TMD (if present), ICD (if present)	Amino acid sequence
Seq 01	VEGFR2 ECD, VEGFR2 TMD, NTEVp[1]	ASVGLPSVSLDLPRLSIQKDILTIKANTTLQITCRGQRDLDWLWPNN<br>QSGSEQRVEVTECSDGLFCKTLTIPKVIGNDTGAYKCFYRETDLAS<br>VIYVYVQDYRSPFIASVSDQHGVVYITENKNKTVVIPCLGSISNLNVS<br>LCARYPEKRFVPDGNRISWDSKKGFTIPSYMISYAGMVFCEAKIND<br>ESYQSIMYIVVVVGYRIYDVVLSPSHGIELSVGEKLVLNCTARTELNV<br>GIDFNWEYPSSKHQHKKLVNRDLKTQSGSEMKKFLSTLTIDGVTRS<br>DQGLYTCAASSGLMTKKNSTFVRVHEKPFVAFGSGMESLVEATVG<br>ERVRIPAKYLGYPPPEIKWYKNGIPLESNHTIKAGHVLTIMEVSERDT<br>GNYTVILTNPISKEKQSHVVSLVVYVPPQIGEKSLISPVDSYQYGTT<br>QTLTCTVYAIPPPHHIHWYWQLEEECANEPSQAVSVTNPYPCEEW<br>RSVEDFQGGNKIEVNKNQFALIEGKNKTVSTLVIQAANVSALYKCEA<br>VNKVGRGERVISFHVTRGPEITLQPDMQPTEQESVSLWCTADRST<br>FENLTWYKLGPQPLPIHVGELPTPVCKNLDTLWKLNATMFSNSTND<br>ILIMELKNASLQDQGDYVCLAQDRKTKKRHCVVRQLTVLERVAPTIT<br>GNLENQTTSIGESIEVSCTASGNPPPQIMWFKDNETLVEDSGIVLKD<br>GNRNLTIRRVRKEDEGLYTCQACSVLGCAKVEAFFIIEGAQEKTNLE<br>IIILVGTAVIAMFFWLLLVIILRTVKGAESLFKGPRDYNPISSTICHLTN<br>ESDGHTTSLYGIGFGPFIITNKHLFRRNNGTLLVQSLHGVFKVKNTT<br>TLQQSLIDGRDMIIIRMPKDFPPFPQKLKFREPQREERICLVTTNFQT
Seq 02	VEGFR1 ECD, VEGFR1 TMD, CTEVp[2]	SKLKDPELSLKGTQHIMQAGQTLHLQCRGEAAHKWSLPEMVSKES<br>ERLSITKSACGRNGKQFCSTLTLNTAQANHTGFYSCKYLAVPTSKK<br>KETESAIYIFISDTGRPFVEMYSEIPEIIHMTEGRELVIPCRVTSPNITV<br>TLKKFPLDTLIPDGKRIIWDSRKGFIISNATYKEIGLLTCEATVNGHLY<br>KTNYLTHRQTNTIIDVQISTPRPVKLLRGHTLVLNCTATTPLNTRVQ<br>MTWSYPDEKNKRASVRRRIDQSNSHANIFYSVLTIDKMQNKDKGLY<br>TCRVRSGPSFKSVNTSVHIYDKAFITVKHRKQQVLETVAGKRSYRL<br>SMKVKAFPSPEVVWLKDGLPATEKSARYLTRGYSLIIKDVTEEDAG<br>NYTILLSIKQSNVFKNLTATLIVNVKPQIYEKAVSSFPDPALYPLGSR<br>QILTCTAYGIPQPTIKWFWHPCNHNHSEARCDFCSNNEESFILDAD<br>SNMGNRIESITQRMAIIEGKNKMASTLVVADSRISGIYICIASNKVGTV<br>GRNISFYITDVPNGFHVNLEKMPTEGEDLKLSCTVNKFLYRDVTWIL<br>LRTVNNRTMHYSISKQKMAITKEHSITLNLTIMNVSLQDSGTYACRA<br>RNVYTGEEILQKKEITIRDQEAPYLLRNLSDHTVAISSSTTLDCHANG<br>VPEPQITWFKNNHKIQQEPGIILGPGSSTLFIERVTEEDEGVYHCKA<br>TNQKGSVESSAYLTVQGTSDKSNLELITLTCTCVAATLFWLLLTLFIR<br>KMKRGAKSMSSMVSDTSCTFPSSDGIFWKHWIQTKDGQCGSPLV<br>STRDGFIVGIHSASNFTNTNNYFTSVPKNFMELKTNQEAQQWVSG<br>WRLNADSVLWGGHKVFMVKPEEPFQPVKEATQLMN
Seq 03	VEGFA121 (monomer)	APMAEGGGQNHHEVVKFMDVYQRSYCHPIETLVDIFQEYPDEIEYI<br>FKPSCVPLMRCGGCCNDEGLECVPTEESNITMQIMRIKPHQGQHIG<br>EMSFLQHNKCECRPKKDRARQEKCDKPRR
Seq 04	VEGFA165 (monomer)	APMAEGGGQNHHEVVKFMDVYQRSYCHPIETLVDIFQEYPDEIEYI<br>FKPSCVPLMRCGGCCNDEGLECVPTEESNITMQIMRIKPHQGQHIG<br>EMSFLQHNKCECRPKKDRARQENPCGPCSERRKHLFVQDPQTCK<br>CSCKNTDSRCKARQLELNERTCRCDKPRR

Seq 05	IL-10Rb ECD, IL-10Rb TMD, NTEVp[1]	MVPPPENVRMNSVNFKNILQWESPAFAKGNLTFTAQYLSYRIFQDK CMNTTLTECDFSSLSKYGDHTLRVRAEFADEHSDWVNITFCPVDDT IIGPPGMQVEVLADSLHMRFLAPKIENEYETWTMKNVYNSWTYNVQ YWKNGTDEKFQITPQYDFEVLRNLEPWTTYCVQVRGFLPDRNKAG EWSEPVCEQTTHDETVPSWMVAVILMASVFMVCLALLGCFALLWC VYKKGAESLFKGPRDYNPISSTICHLTNESDGHTTSLYGIGFGPFIIT NKHLFRRNNGTLLVQSLHGVFKVKNTTTLQQSLIDGRDMIIIRMPKD FPPFPQKLKFREPQREERICLVTTNFQT
Seq 06	IL-10Ra ECD, IL-10Ra TMD, CTEVp[2]	HGTELPSPPSVWFEAEFFHHILHWTPIPNQSESTCYEVALLRYGIES WNSISNCSQTLSYDLTAVTLDLYHSNGYRARVRAVDGSRHSNWTV TNTRFSVDEVTLTVGSVNLEIHNGFILGKIQLPRPKMAPANDTYESIF SHFREYEIAIRKVPGNFTFTHKKVKHENFSLLTSGEVGEFCVQVKPS VASRSNKGMWSKEECISLTRQYFTVTNVIIFFAFVLLLSGALAYCLAL QLYVRRRKKGAKSMSSMVSDTSCTFPSSDGIFWKHWIQTKDGQC GSPLVSTRDGFIVGIHSASNFTNTNNYFTSVPKNFMELKTNQEAQQ WVSGWRLNADSVLWGGHKVFMVKPEEPFQPVKEATQLMN
Seq 07	IL-10 (monomer)	SPGQGTQSENSCTHFPGNLPNMLRDLRDAFSRVKTFFQMKDQLD NLLLKESLLEDFKGYLGCQALSEMIQFYLEEVMPQAENQDPDIKAH VNSLGENLKTLRLRLRRCHRFLPCENKSKAVEQVKNAFNKLQEKGI YKAMSEFDIFINYIEAYMTMKIRN
Seq 08	IL-10Rb ECD, CD28 TMD, NTEVp[1]	MVPPPENVRMNSVNFKNILQWESPAFAKGNLTFTAQYLSYRIFQDK CMNTTLTECDFSSLSKYGDHTLRVRAEFADEHSDWVNITFCPVDDT IIGPPGMQVEVLADSLHMRFLAPKIENEYETWTMKNVYNSWTYNVQ YWKNGTDEKFQITPQYDFEVLRNLEPWTTYCVQVRGFLPDRNKAG EWSEPVCEQTTHDETVPSTGLVVVAGVLFCYGLLVTVALCVGGGS GGGAESLFKGPRDYNPISSTICHLTNESDGHTTSLYGIGFGPFIITNK HLFRRNNGTLLVQSLHGVFKVKNTTTLQQSLIDGRDMIIIRMPKDFP PFPQKLKFREPQREERICLVTTNFQT
Seq 09	IL-10Ra ECD, CD28 TMD, CTEVp[2]	HGTELPSPPSVWFEAEFFHHILHWTPIPNQSESTCYEVALLRYGIES WNSISNCSQTLSYDLTAVTLDLYHSNGYRARVRAVDGSRHSNWTV TNTRFSVDEVTLTVGSVNLEIHNGFILGKIQLPRPKMAPANDTYESIF SHFREYEIAIRKVPGNFTFTHKKVKHENFSLLTSGEVGEFCVQVKPS VASRSNKGMWSKEECISLTRQYFTVTNTGLVVVAGVLFCYGLLVTV ALCVGGGSGGGAKSMSSMVSDTSCTFPSSDGIFWKHWIQTKDGQ CGSPLVSTRDGFIVGIHSASNFTNTNNYFTSVPKNFMELKTNQEAQ QWVSGWRLNADSVLWGGHKVFMVKPEEPFQPVKEATQLMN
Seq 10	VEGFR1 ECD	SKLKDPELSLKGTQHIMQAGQTLHLQCRGEAAHKWSLPEMVSKES ERLSITKSACGRNGKQFCSTLTLNTAQANHTGFYSCKYLAVPTSKK KETESAIYIFISDTGRPFVEMYSEIPEIIHMTEGRELVIPCRVTSPNITV TLKKFPLDTLIPDGKRIIWDSRKGFIISNATYKEIGLLTCEATVNGHLY KTNYLTHRQTNTIIDVQISTPRPVKLLRGHTLVLNCTATTPLNTRVQ MTWSYPDEKNKRASVRRRIDQSNSHANIFYSVLTIDKMQNKDKGLY TCRVRSGPSFKSVNTSVHIYDKAFITVKHRKQQVLETVAGKRSYRL SMKVKAFPSPEVVWLKDGLPATEKSARYLTRGYSLIIKDVTEEDAG NYTILLSIKQSNVFKNLTATLIVNVKPQIYEKAVSSFPDPALYPLGSR QILTCTAYGIPQPTIKWFWHPCNHNHSEARCDFCSNNEESFILDAD SNMGNRIESITQRMAIIEGKNKMASTLVVADSRISGIYICIASNKVGTV GRNISFYITDVPNGFHVNLEKMPTEGEDLKLSCTVNKFLYRDVTWIL LRTVNNRTMHYSISKQKMAITKEHSITLNLTIMNVSLQDSGTYACRA RNVYTGEEILQKKEITIRDQEAPYLLRNLSDHTVAISSSTTLDCHANG VPEPQITWFKNNHKIQQEPGIILGPGSSTLFIERVTEEDEGVYHCKA TNQKGSVESSAYLTVQGTSDKSNLE

Seq 11	VEGFR2 ECD	ASVGLPSVSLDLPRLSIQKDILTIKANTTLQITCRGQRDLDWLWPNN QSGSEQRVEVTECSDGLFCKTLTIPKVIGNDTGAYKCFYRETDLAS VIYVYVQDYRSPFIASVSDQHGVVYITENKNKTVVIPCLGSISNLNVS LCARYPEKRFVPDGNRISWDSKKGFTIPSYMISYAGMVFCEAKIND ESYQSIMYIVVVVGYRIYDVVLSPSHGIELSVGEKLVLNCTARTELNV GIDFNWEYPSSKHQHKKLVNRDLKTQSGSEMKKFLSTLTIDGVTRS DQGLYTCAASSGLMTKKNSTFVRVHEKPFVAFGSGMESLVEATVG ERVRIPAKYLGYPPPEIKWYKNGIPLESNHTIKAGHVLTIMEVSERDT GNYTVILTNPISKEKQSHVVSLVVYVPPQIGEKSLISPVDSYQYGTT QTLTCTVYAIPPPHHIHWYWQLEEECANEPSQAVSVTNPYPCEEW RSVEDFQGGNKIEVNKNQFALIEGKNKTVSTLVIQAANVSALYKCEA VNKVGRGERVISFHVTRGPEITLQPDMQPTEQESVSLWCTADRST FENLTWYKLGPQPLPIHVGELPTPVCKNLDTLWKLNATMFSNSTND ILIMELKNASLQDQGDYVCLAQDRKTKKRHCVVRQLTVLERVAPTIT GNLENQTTSIGESIEVSCTASGNPPPQIMWFKDNETLVEDSGIVLKD GNRNLTIRRVRKEDEGLYTCQACSVLGCAKVEAFFIIEGAQEKTNLE
Seq 12	TNFR1 ECD	LVPHLGDREKRDSVCPQGKYIHPQNNSICCTKCHKGTYLYNDCPG PGQDTDCRECESGSFTASENHLRHCLSCSKCRKEMGQVEISSCTV DRDTVCGCRKNQYRHYWSENLFQCFNCSLCLNGTVHLSCQEKQN TVCTCHAGFFLRENECVSCSNCKKSLECTKLCLPQIENVKGTEDSG TT
Seq 13	TNFR2 ECD	LPAQVAFTPYAPEPGSTCRLREYYDQTAQMCCSKCSPGQHAKVFC TKTSDTVCDSCEDSTYTQLWNWVPECLSCGSRCSSDQVETQACT REQNRICTCRPGWYCALSKQEGCRLCAPLRKCRPGFGVARPGTE TSDVVCKPCAPGTFSNTTSSTDICRPHQICNVVAIPGNASMDAVCT STSPTRSMAPGAVHLPQPVSTRSQHTQPTPEPSTAPSTSFLLPMG PSPPAEGSTGD
Seq 14	TNF (monomer)	VRSSSRTPSDKPVAHVVANPQAEGQLQWLNRRANALLANGVELR DNQLVVPSEGLYLIYSQVLFKGQGCPSTHVLLTHTISRIAVSYQTKV NLLSAIKSPCQRETPEGAEAKPWYEPIYLGGVFQLEKGDRLSAEINR PDYLDFAESGQVYFGIIAL
Seq 15	IL-10Ra ECD	HGTELPSPPSVWFEAEFFHHILHWTPIPNQSESTCYEVALLRYGIES WNSISNCSQTLSYDLTAVTLDLYHSNGYRARVRAVDGSRHSNWTV TNTRFSVDEVTLTVGSVNLEIHNGFILGKIQLPRPKMAPANDTYESIF SHFREYEIAIRKVPGNFTFTHKKVKHENFSLLTSGEVGEFCVQVKPS VASRSNKGMWSKEECISLTRQYFTVTN
Seq 16	IL-10Rb ECD	MVPPPENVRMNSVNFKNILQWESPAFAKGNLTFTAQYLSYRIFQDK CMNTTLTECDFSSLSKYGDHTLRVRAEFADEHSDWVNITFCPVDDT IIGPPGMQVEVLADSLHMRFLAPKIENEYETWTMKNVYNSWTYNVQ YWKNGTDEKFQITPQYDFEVLRNLEPWTTYCVQVRGFLPDRNKAG EWSEPVCEQTTHDETVPS
Seq 17	TGFβR1 ECD	LQCFCHLCTKDNFTCVTDGLCFVSVTETTDKVIHNSMCIAEIDLIPRD RPFVCAPSSKTGSVTTTYCCNQDHCNKIELPTTVKSSPGLGPVEL
Seq 18	TGFβR2 ECD	TIPPHVQKSVNNDMIVTDNNGAVKFPQLCKFCDVRFSTCDNQKSC MSNCSITSICEKPQEVCVAVWRKNDENITLETVCHDPKLPYHDFILE DAASPKCIMKEKKKPGETFFMCSCSSDECNDNIIFSEEYNTSNPDLL LVIFQ
Seq 19	TGFβ1 (monomer)	ALDTNYCFSSTEKNCCVRQLYIDFRKDLGWKWIHEPKGYHANFCL GPCPYIWSLDTQYSKVLALYNQHNPGASAAPCCVPQALEPLPIVYY VGRKPKVEQLSNMIVRSCKCS
Seq 20	VEGFR1 TMD	LITLTCTCVAATLFWLLLTLFI
Seq 21	VEGFR2 TMD	IIILVGTAVIAMFFWLLLVII
Seq 22	TNFR1 TMD	VLLPLVIFFGLCLLSLLFIGL
Seq 23	TNFR2 TMD	FALPVGLIVGVTALGLLIIGVVNCVIMTQV

Seq 24	IL-10Ra TMD	VIIFFAFVLLLSGALAYCLAL
Seq 25	IL-10Rb TMD	WMVAVILMASVFMVCLALLGCF
Seq 26	TGFBR1 TMD	AAVIAGPVCFVCISLMLMVYI
Seq 27	TGFBR2 TMD	VTGISLLPPLGVAISVIIIFY
Seq 28	Murine CD28 TMD	LVVVAGVLFCYGLLVTVALCV

1. The NTEVp variant used has an H75S mutation from wildtype
2. The CTEVp variant used has an L190K mutation from wildtype

Supplementary Table 3: PREDDIMER TMD dimer quality scores

TMD 1	TMD 2	Fscor
VEGFR1	VEGFR1	2.838
VEGFR1	VEGFR2	2.096
VEGFR2	VEGFR1	2.449
VEGFR2	VEGFR2	2.351
VEGFR1	CD28	2.645
CD28	VEGFR1	2.648
VEGFR2	CD28	2.448
CD28	VEGFR2	2.479
TNFR1	TNFR1	3.267
TNFR1	TNFR2	3.174
TNFR2	TNFR1	2.968
TNFR2	TNFR2	2.805
TNFR1	CD28	2.785
CD28	TNFR1	2.93
TNFR2	CD28	2.864
CD28	TNFR2	2.636
IL-10Ra	IL-10Ra	2.69
IL-10Ra	IL-10Rb	2.713
IL-10Rb	IL-10Ra	2.668
IL-10Rb	IL-10Rb	2.779
IL-10Ra	CD28	2.719
CD28	IL-10Ra	2.831
IL-10Rb	CD28	2.317
CD28	IL-10Rb	2.436
TGFBR1	TGFBR1	2.447
TGFBR1	TGFBR2	3.105
TGFBR2	TGFBR1	3.345
TGFBR2	TGFBR2	4.334
TGFBR1	CD28	2.815
CD28	TGFBR1	2.759
TGFBR2	CD28	3.549
CD28	TGFBR2	3.618
CD28	CD28	2.723
